# Draft genome sequence of *Bacillus cereus* MHES3, a potential plant probiotic isolated from the tomato rhizosphere in a saline-prone ecosystem of Bangladesh

**DOI:** 10.1128/mra.01060-26

**Published:** 2026-10-27

**Authors:** Rifat Islam, Md. Nayeem Hossain, Md. Nazmul Hannan, Kamrun Nahar, M. Nazmul Hoque, Md. Mizanur Rahman, Md. Manjurul Haque

**Affiliations:** 1 Department of Environmental Science, Faculty of Agriculture, Gazipur Agricultural University 198780, Gazipur, Bangladesh; 2 Biotechnology Division, Bangladesh Agricultural Research Institute, Gazipur, Bangladesh; 3 Molecular Biology and Bioinformatics Laboratory, Department of Gynecology, Obstetrics and Reproductive Health, Faculty of Veterinary Medicine and Animal Science, Gazipur Agricultural University 198780, Gazipur, Bangladesh; 4 Department of Soil Science, Faculty of Agriculture, Gazipur Agricultural University, Gazipur, Bangladesh; University of Manitoba, Winnipeg, Canada

**Keywords:** *Bacillus cereus*, rhizosphere, genome sequencing, salinity stress, heavy-metal resistance

## Abstract

We report the draft genome of *Bacillus cereus* MHES3, a stress-tolerant plant probiotic candidate from saline tomato rhizosphere soil in Bangladesh. The 5.6-Mb genome (65 contigs; 35.0% GC; 5,507 CDS) harbors genes for growth promotion, nutrient acquisition, stress adaptation, and heavy-metal resistance, supporting future climate-resilient agriculture and bioremediation research.

## ANNOUNCEMENT

*Bacillus cereus* is a gram-positive, spore-forming bacterium that includes plant-associated strains with diverse plant growth-promoting activities (1, 2). Importantly, some *B. cereus* strains exhibit tolerance to environmental stresses, including salinity, drought, temperature fluctuations, and toxic heavy metals, highlighting their potential relevance to climate-resilient and sustainable agriculture (3, 4). Therefore, whole-genome sequencing provides valuable insights into the genetic basis of plant growth promotion and environmental stress adaptation and facilitates the identification of functional traits associated with the environmental and agricultural potential of plant-associated *B. cereus* strains (2, 5).

*B. cereus* strain MHES3 was isolated from the rhizosphere of a tomato plant (*Solanum lycopersicum* L.) grown in a saline-prone ecosystem in Mongla, Bagerhat District of Bangladesh. Approximately 1 g of rhizosphere soil was suspended in 9 mL of sterile 0.85% NaCl and serially diluted from 10⁻¹ to 10⁻⁷, and 100 µL aliquots were plated in triplicate onto yeast extract–peptone agar. Plates were incubated at 28°C for 48 h. A morphologically distinct colony was purified through three successive rounds of streaking and preserved in 20% glycerol at −80°C.

Genomic DNA was extracted using the GeneJET Genomic DNA Purification Kit (Thermo Fisher Scientific, USA) according to the manufacturer’s instructions. DNA quality and quantity were assessed using a NanoDrop 2000 spectrophotometer and a Qubit 4.0 fluorometer, respectively. A sequencing library was prepared from 1 ng of genomic DNA using the Nextera DNA Flex Library Preparation Kit (Illumina) according to the manufacturer’s protocol. Sequencing was performed on an Illumina MiSeq platform, generating 713,876 paired-end pairs (1,427,752 reads in total, counting both R1 and R2) with a mean read length of 266 bp (*σ* = 62.4), totaling 380.1 Mbp (6). Raw reads were assessed using FastQC v0.12.1 (7) and trimmed using fastp v0.22.0 (8). The quality-filtered reads were assembled *de novo* using SPAdes v4.2.0 (9). Genome completeness and contamination were evaluated using CheckM v1.2.5 (10). Taxonomic identity was assessed by average nucleotide identity (ANI) analysis against the *B. cereus* type strain ATCC 14579, with MHES3 showing 97.12% ANI, supporting its assignment to *B. cereus*. The genome was annotated using the NCBI Prokaryotic Genome Annotation Pipeline (PGAP) v6.11 (9). Default parameters were used unless otherwise stated.

The draft genome assembly comprises 65 contigs (50 scaffolds), totaling 5.6 Mb, with an N50 value of 173.2 kb, a GC content of 35.0%, and approximately 68× sequencing coverage. CheckM estimated the genome to be 99.18% complete, with 0.06% contamination. PGAP identified 5,810 genes, including 5,507 protein-coding sequences, 86 tRNAs, and 189 pseudogenes. Plasmid screening using PlasmidFinder (11) detected no plasmid replicons. Further analysis identified genes associated with functions relevant to plant-associated lifestyles, including siderophore biosynthesis and transport (*dhb* operon and *fep*/*fhu* systems), phosphate acquisition (*pstABCS* and *phoA*), potassium uptake (*kdpABC*), acetoin and 2,3-butanediol biosynthesis (*alsS* and *budA–C*), urease production (*ureABCDEFG*), and chitin degradation (2, 12, 13). The genome also contained genes associated with resistance to arsenic (*arsB*, *arsC*, and *arsR*), chromate (*chrA*), copper (*copA*, *copC*, *copZ*, *cueO*, *cueR*, and *cutC*), and zinc, cobalt, and cadmium (*czcD*, *zntA*, *zntB*, and *zntR*) (14). The azoreductase *azoR* and the multicopper oxidase *cueO*, alongside broad-specificity flavin/quinone reductases (*nuo*, *wrbA*, and *qor*) of uncertain dye specificity, were also identified (15). These genomic features provide a foundation for further phenotypic and functional studies to explore the potential application of MHES3 in climate-resilient agriculture, sustainable crop production, and microbe-assisted remediation of heavy-metal-contaminated environments (Table 1).

**TABLE 1:** Genomic features of *B. cereus* strain MHES3 isolated from tomato rhizosphere in Bagerhat, Bangladesh

Genome features	*B. cereus* MHES3
GenBank accession no.	JCBASU000000000
BioProject accession no.	PRJNA1497355
BioSample accession no.	SAMN61801243
SRA accession no.	SRR39712100
Collection date (yr-mo-day)	2021-08-03
Sequencing platform	Illumina MiSeq
Library layout	Paired end
Illumina read length (bp)	266 (mean, *σ* = 62.4)
Total reads	1,427,752 (counting both R1 and R2)
Total bases (Mbp)	380.1
Assembled genome size (Mb)	5.6 Mb (5.5 Mb ungapped)
GC content (%)	35.0
Coverage (×)	68
Genome completeness (%)	99.18
Contamination (%)	0.06
N50 value (bp)	173,200 (contig)/355,800 (scaffold)
Number of contigs (scaffolds)	65 (50)
Genes (total)	5,810
Coding sequences (protein coding)	5,507
tRNAs	86
tmRNA	1
rRNA	22
ncRNA	23
CRISPR array	1
Pseudogenes (total)	189
ANI to *B. cereus* ATCC 14579 (%)	97.12
Plasmid replicons	None detected

## Data Availability

The whole-genome shotgun project has been deposited in GenBank under accession no. JCBASU000000000. The version described in this paper is JCBASU010000000. Raw sequencing reads are available in the NCBI Sequence Read Archive under accession no. SRR39712100. The genome sequence is associated with BioProject PRJNA1497355 (“Development of Climate-Smart Bacterial Biofertilizers”) and BioSample SAMN61801243.

## AFTER EPUB

*[This article was published on 27 October 2026 with errors in the author byline and affiliations. The byline and affiliations were corrected in the current version, posted on 30 October 2026.]*
